# Trigeminal Neuralgia Secondary to Herpes Simplex Virus Type 1 Infection Treated With Oral Acyclovir

**DOI:** 10.7759/cureus.54128

**Published:** 2024-02-13

**Authors:** Amal J Alfaifi, Ohood S Wasli, Thamer M Almalki, Ahmad Y Alqassim

**Affiliations:** 1 Family Medicine, Jazan Health Cluster, Jazan, SAU; 2 Family Medicine, Armed Forces Hospital, Jazan, SAU; 3 Faculty of Medicine, Jazan University, Jazan, SAU; 4 Family and Community Medicine, Faculty of Medicine, Jazan University, Jazan, SAU

**Keywords:** trigeminal neuralgia, ‏antiviral therapy, ‏facial pain, ‏acyclovir, ‏herpes simplex virus type 1

## Abstract

Trigeminal neuralgia (TN) is characterized by episodic electric, shock-like facial pain. Though often idiopathic, herpes simplex virus type 1 (HSV-1) reactivation can rarely cause symptomatic TN. We report the case of a 30-year-old woman who developed oral HSV-1 lesions followed by right-sided TN pain. MRI of the brain did not reveal neurovascular compression. TN pain completely resolved with oral acyclovir treatment alone, without anticonvulsants. This highlights the importance of considering atypical etiologies such as HSV-1 reactivation in TN evaluation. Early antiviral therapy may treat underlying inflammation and provide sustained symptom relief in HSV-associated TN.

## Introduction

Trigeminal neuralgia (TN) is a chronic pain condition characterized by sudden, severe, electric shock-like facial pain along the distributions of the trigeminal nerve [1]. It has an estimated incidence of 4-5 per 100,000 people annually and is more common among women and people over 50 years of age [2]. The exact cause of TN is unknown, but it is thought to be related to compression of the trigeminal nerve root near the brainstem by blood vessels or lesions [3]. The diagnosis of TN is based on a history of characteristic pain attacks that are consistent with specific widely accepted criteria for the diagnosis [4]. Treatments aim to reduce nerve irritation through medications such as anticonvulsants or surgical procedures such as microvascular decompression [5].

Herpes simplex virus type 1 (HSV-1) is a highly prevalent infection worldwide, with an estimated 3.7 billion people under the age of 50 years infected globally [6]. HSV-1 is typically transmitted during childhood through direct contact and establishes life-long latency in nerve ganglia [7]. While HSV-1 often causes orolabial lesions, it can also rarely present with neurological complications such as meningitis, encephalitis, and TN [8]. Symptoms of a primary orolabial infection occur between three days and one week after the exposure [9]. The proposed mechanism involves HSV-1-related inflammation affecting the trigeminal ganglia and sensory nerve roots [10].

HSV-1 spreads from the oral pharynx to the trigeminal ganglia, where a latent HSV-1 infection is established. Cold sores at the mucocutaneous junction of the lip are the typical manifestation of recurrent HSV-1 [11].

Standard treatments for classical TN may not be sufficient for symptomatic TN caused by viral inflammation. Case reports have described successful treatment of HSV-1-related TN using antiviral agents such as acyclovir rather than anticonvulsants [12]. Here, we present a case of TN symptoms associated with oral HSV-1 lesions successfully treated with oral acyclovir, highlighting the importance of considering alternative etiologies of TN.

## Case presentation

A 30-year-old previously healthy female presented with a three-day history of redness, mild swelling, and itching of the left upper lip. This was followed by the development of painful fluid-filled blisters localized to a 2 x 2 cm area of the upper lip. A review of systems was negative for fever, malaise, or lymphadenopathy, and there was no history of associated ear pain, hair loss, or other accompanying symptoms. She had no prior episodes of HSV-1 infection, other medical conditions, or medications.

**Figure 1:**
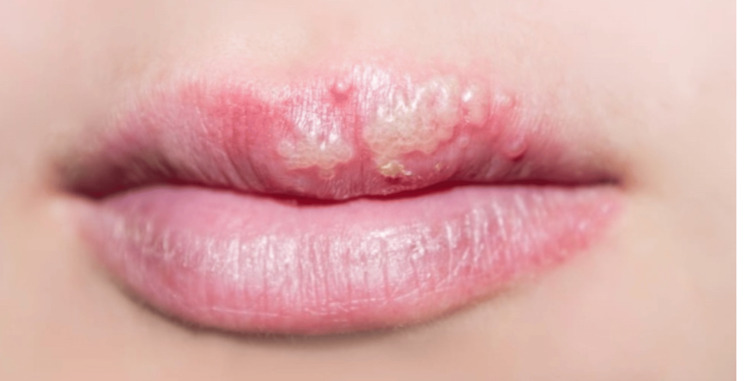
Herpes simplex lesions on the upper lip.

On examination, vital signs were normal. Multiple small painful vesicles were noted on the upper lip, with surrounding normal skin (Figure 1). The lesions progressively increased in size and number over the next two days, spreading along the left upper lip and measuring approximately 2 x 3 cm in total surface area.

Polymerase chain reaction and viral culture of the vesicular fluid were positive for HSV-1. Serum HSV-1 IgG was also positive, indicating prior exposure to HSV-1, but did not confirm acute reactivation. MRI of the brain and brainstem did not show any masses or vascular compression that could explain TN.

The patient was started on topical acyclovir five times daily on day three of skin lesion onset. After five days, the vesicles crusted over, and healing was observed. Three days after the skin lesions first appeared, she developed severe left-sided facial and jaw pain in the V2/V3 dermatomes. The pain was described as sharp, electric shock-like, and triggered by light touch, brushing teeth, eating, and exposure to wind. Based on the distribution and quality, a clinical diagnosis of TN was made.

Oral acyclovir 800 mg five times daily was initiated alongside the topical treatment. Over the next seven days, the intensity and frequency of TN pain attacks gradually improved. By day 14 since lesion onset, she had complete resolution of HSV-1 lesions and TN symptoms. At the three-month follow-up, she remained free of further HSV-1 outbreaks and TN pain.

## Discussion

The pathogenesis of HSV-1-associated TN is thought to involve inflammation and irritation of the trigeminal ganglia and sensory nerve roots [10]. Reactivation of latent HSV-1 infection leads to viral replication in trigeminal ganglion cells, prompting an immune response and localized nerve damage [12]. This nerve irritation presents clinically as trigeminal distribution pain with a similar phenotype to classical TN. However, HSV-1-associated pain may have a more superficial and diffuse quality compared to the sharp, electric paroxysms in areas of trigeminal nerve compression [13].

First-line treatment for classical TN without an underlying etiology is anticonvulsant medications such as carbamazepine or oxcarbazepine [14]. For TN secondary to viral inflammation, antiviral agents may treat the underlying condition and provide symptomatic relief by reducing nerve irritation [15]. Our case demonstrated complete resolution of trigeminal pain with oral acyclovir, without needing anticonvulsants. However, it is difficult to conclusively ascertain whether the improvement in TN symptoms was due specifically to oral acyclovir, topical acyclovir alone, the natural disease course, or a combination of factors based on this case report alone without a control group for comparison. This suggests antiviral therapy alone may be sufficient in some cases of HSV-related TN. Early initiation of antivirals, as in our case, could potentially limit nerve damage and prevent recurring bouts of trigeminal pain.

There are limitations in drawing definitive conclusions from a single case report. While promising, the efficacy of antiviral treatment instead of conventional anticonvulsants requires further investigation in controlled trials. However, our case findings align with other reported cases of effective acyclovir or valacyclovir use for suspected HSV-associated TN [15-17].

## Conclusions

This case highlights the importance of considering atypical etiologies such as HSV-1 reactivation in patients presenting with TN features. A thorough history and directed diagnostic testing can identify contributing factors. Prompt antiviral therapy may provide both symptomatic relief and reduce the risk of subsequent neuralgia attacks in trigeminal pain secondary to viral inflammation. While promising, controlled studies are needed to better determine the efficacy of antiviral therapy compared to natural history or placebo for HSV-associated TN.
